# Recovery from general anaesthesia in the elderly patient: sevoflurane vs propofol

**DOI:** 10.1186/1471-2318-11-S1-A3

**Published:** 2011-08-24

**Authors:** B Bastianini, L Vessicchio, F Grassia, B Lettieri

**Affiliations:** 1 Department of Anaesthesia, Surgical and Emergency Science, Second University of Naples, Italy

## Background

Pharmacokinetics of anaesthetic drugs is greatly influenced by paraphysiological modifications due to advanced age:

– Slower drug metabolism

– Decrease in TBW

– Increase in LBM/FBM

– Decrease in kidney function

This leads to the need to use short half-life drugs, to prevent drug stacking phenomena. Both sevoflurane and propofol meet these requirements.

## Materials and methods

This study compares recovery times from general anaesthesia between the selected drugs in patients over 65yo who underwent surgery.

In 3 months 41 patients were selected, similar in age (65-78yo), sex and ASA class, undergoing elective surgery. Patients with lung, liver, kidney, brain and coronaric dysfunctions with ASA III-IV were excluded from the study.

In this single-blind, prospectic study patients were randomized in 2 groups: patients in P-group were treated with propofol (5-8 mg/kg/h), while patients in S-group were treated with sevoflurane (1-2%).

Dosage was managed so that CF and AP wouldn’t shift more than 20% their basal values. All patients were pre-treated with midazolam (0.03mg/kg), fentanyl (1μg/kg) and atropine (up to 0.01ng/kg). Anaesthesia was induced using propofol (2mg/kg) and cisatracurium (0.2mg/kg). Patients were intubated and ventilated using O_2_ (33%) and N_2_O (66%). Analgesia during surgery was achieved using fentanyl (1μg/kg) 60min after induction and then using boli every 45min. Parameters monitored for each patient were: AP, CF, ECG, PO_2_, ET CO_2,_ BIS and diuresis. Times for extubation, recovery room monitoring, eye opening and oriented motory and verbal response were recorded. MMSE was also taken.

## Results

41 Patients were selected (table 1), age 65-78, ASA II; groups were homogeneous in type and duration of surgery (120±67min). Induction and intra-surgery dosages of drugs were similar between patients, as well as 2-hours post-operation pain-score. Extubation was quicker in S-group (6.8±4min) than in P-group (9.8±5min). BIS system score has shown quicker recover from sleep in S-group (table 2). Simple commands execution (eye opening, hand movement) and time-space orientation recovery (saying one’s own name and birthday) were quicker in the S-group as well (table 3). Discharge times from recovery room were similar between groups, as were results in MMSE.

**Table 1:** Prospectic, randomized study, patients selected Patients total 41

S	P
21 pz. (9M/12F)	20 pz. (10M/10F)

**Table 2:** recovery after sevoflurane or propofol suspension

	S	P
Extubation (min)	6.8±4	9.8±5
Eye Opening	5.9±2	9.3±2
Punch (orientated motory response)	10.2±2	14±4
Orientated verbal response	12±8	18±5
MMSE	24±4	24±4
Observation time (min)	39±9	40±21
Pain score (2 hours after surgery)	3±2	3±3

**Table 3:** monitored parameters

AP	S	P
Pre-operatory (mmHg)	94±4	94±2
Induction (mmHg)	116±5	100±8
Incision (mmHg)	80±6	109±9.5
Maintenance (mmHg)	83±2	74±7
Recovery (mmHg)	105±5	107±11

**CF**	**S**	**P**

Pre-operatory (b/min)	72±7	72±7
Intubation (b/min)	100±10	87±6
Incision (b/min)	81±9	88±11
Maintenance (b/min)	78±5	67±13
Recovery (b/min)	88±7	90±8

## Conclusions

Sevoflurane shows the lowest periferic blood solubility and the lowest partition ratio amongst inhaled anaesthetics. This grants both quick induction and recovery. These properties associated with better hemodynamic stability during maintenance of general anaesthesia make this drug favorable for the elderly patient, who often has deficitary cardiac homeostatic mechanisms, both due to advanced age and to other associated diseases.
